# Four-year analysis of high-risk human papillomavirus infection among women in rural areas of Nyingchi City, Tibet

**DOI:** 10.3389/fpubh.2023.1251440

**Published:** 2023-09-20

**Authors:** Jianqi Li, Xiaojie Li, Xiujie Sheng

**Affiliations:** ^1^Department of Obstetrics and Gynecology, Guangdong Provincial Key Laboratory of Major Obstetric Diseases, Guangdong Provincial Clinical Research Center for Obstetrics and Gynecology, Guangdong-Hong Kong-Macao Greater Bay Area Higher Education Joint Laboratory of Maternal-Fetal Medicine, The Third Affiliated Hospital of Guangzhou Medical University, Guangzhou, China; ^2^Department of Obstetrics and Gynecology, People’s Hospital of Bomi, Nyingchi, Tibet, China

**Keywords:** HR-HPV, HR-HPV subtypes, rural women, Qinghai-Tibet plateau, Tibet (China)

## Abstract

**Purpose:**

This study aims to address the existing data gap regarding the status of high-risk human papillomavirus (HR-HPV) infection and the distribution of HR-HPV subtypes among women in rural areas of Nyingchi City, Tibet. The research objectives include providing insights for HPV vaccine development.

**Methods:**

The research collected data from two rounds of cancer screening conducted among rural women in Nyingchi City, Tibet, from 2019 to 2022. HR-HPV subtype gene detection was performed using the PCR fluorescence method on the collected samples. And then analyzed the HR-HPV infection rate among rural women in Nyingchi City, Tibet, as well as the infection rate of different HR-HPV subtypes and their distribution across different age groups. A comparison was made between the infection rates of women in rural areas outside the Qinghai-Tibet Plateau and those in Nyingchi City.

**Results:**

A total of 15,687 cases included. The overall HR-HPV infection rate among women in rural areas of Nyingchi City, Tibet, was 13.00% (2040/15,687), which was significantly higher than the rate among women in rural areas outside the Qinghai-Tibet Plateau (7.82% (9,249/118,237); *χ*^2^ = 635.7, *p* < 0.001). The highest HPV infection rate was observed in the 35–39 age group, with a rate of 15.31% (499/3260), which was significantly higher than the rate of 7.22% (1827/25,322) among women in the same age group in rural areas outside the Qinghai-Tibet Plateau (*χ*^2^ = 253.00, *p* < 0.001). The lowest HPV infection rate was found in the 50–54 age group, with a rate of 9.69% (246/2540), which was statistically different from the rate of 8.14% (1,604/19,698) among women in the same age group outside the Qinghai-Tibet Plateau (*χ*^2^ = 17.68, *p* < 0.001). The top three HR-HPV subtypes among women in rural areas of Nyingchi City, Tibet, were HPV52 (20.15%, 411/2040), HPV16 (12.45%, 254/2040), and HPV58 (11.96%, 244/2040). These findings align with the top three HR-HPV subtypes among women in rural areas outside the Qinghai-Tibet Plateau. Furthermore, the top three HR-HPV subtypes among women aged 35–39, 40–44, and 45–49 in rural areas of Nyingchi City, Tibet, were HPV52, HPV16, and HPV58. In conclusion, the HR-HPV infection rate among women in rural areas of Nyingchi City, Tibet, is significantly higher compared to women in rural areas outside the Qinghai-Tibet Plateau, with consistent patterns observed in the distribution of the top three HR-HPV subtypes between the two regions.

## Introduction

In 2018, cervical cancer accounted for approximately 570,000 new cases worldwide, resulting in over 310,000 deaths (1, 2). A comprehensive epidemiological study conducted in 1999 by the International Agency for Research on Cancer involving 22 countries revealed a close association between human papillomavirus (HPV) and 99.7% of cervical cancer cases (3). Persistent infection with high-risk human papillomavirus (HR-HPV) subtypes is the primary cause of cervical precancerous lesions and cervical cancer (4). Notably, 13 HR-HPV subtypes, including HPV types 16, 18, 31, 33, 35, 39, 45, 51, 52, 56, 58, 59, and 68, have been identified as predominantly responsible for cervical cancer (5).

Research on HR-HPV subtype infections among different regions and races in China has been limited to specific provinces or cities in the eastern and central regions, with significant variations observed in the distribution of HR-HPV subtypes across different regions (6–11). Previous studies have reported an overall HR-HPV infection rate of 9.19% among women residing in urban areas of Lhasa, Shigatse, and Nagqu in Tibet, and the most prevalent HR-HPV subtypes include HPV 16, HPV 33, HPV 58, HPV 52, and HPV 31 (12). However, precise data regarding HR-HPV infection among women in rural areas of the Qinghai-Tibet Plateau, including epidemiological data on HR-HPV subtypes, remain lacking.

In addition to the well-established correlation between HR-HPV strains and cervical abnormalities or cervical carcinoma, the noteworthy impact of low-risk human papillomavirus (LR-HPV), particularly HPV 6 and HPV 11, in causing genital warts has attracted considerable attention. The development of HPV vaccines, notably the multi-valent variant, emphasizes the importance of including protection against HPV 6 and HPV 11 (13). However, there is a scarcity of epidemiological data pertaining to LR-HPV, particularly in Qinghai-Tibet Plateau (14–16). This study draws on cervical cancer screening results as its primary data source, concentrating exclusively on HR-HPV strains, while not encompassing pertinent data regarding LR-HPV.

This information is crucial given the limited cross-protection provided by existing HPV vaccines against HR-HPV subtypes prevalent in rural areas of China. Therefore, conducting further research on the prevalence of HR-HPV infection and HR-HPV subtype distribution among women in rural areas of Nyingchi City, Tibet is essential to enhance our understanding of HR-HPV epidemiology and to facilitate the development of domestic HPV vaccines.

### Research objects and methods

Situated in the southeastern part of the Tibet Autonomous Region, Nyingchi City enjoys an average elevation of under 3,000 meters, placing it below higher-altitude areas like Lhasa, Shigatse, Nagqu, and Shannan. This geographical distinction results in a consistent abundance of rainfall and flourishing vegetation throughout the year, earning Nyingchi the epithet “Plateau Jiangnan.” Given the dearth of HPV infection data in Nyingchi City, it becomes imperative to meaningfully compare the HPV infection distribution in this “Plateau Jiangnan” with rural areas outside the Qinghai-Tibet Plateau, especially considering existing data from high-altitude regions in Tibet (14–16).

**Figure 1:**
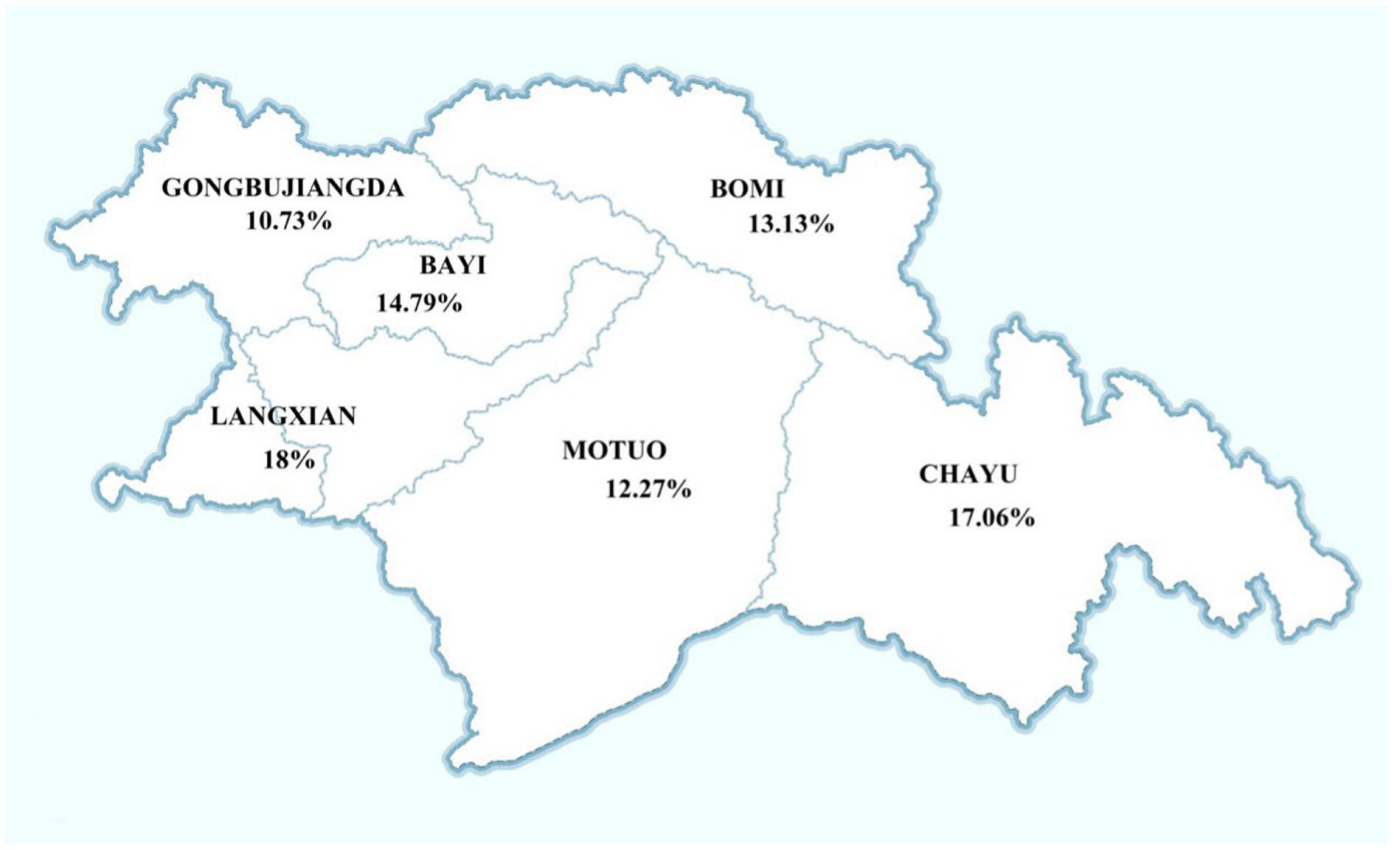
HR-HPV infection rates across various districts in Nyingchi City.

According to the requirements of the national “Screening of Cervical cancer and Breast cancer Program” in China, this study focused on women aged 35–64 with rural household registration and a history of sexual activity. The research collected cervical cancer screening data (HPV results) from Bomi County, Chayu County, Motuo County, GongbuJiangda County, Lang County, and Bayi District in Nyingchi City, Tibet, spanning the period from 2019 to 2022. Inclusion criteria encompassed women aged 35–64 with rural household registration, residing continuously for over 1 year, and willingly participating in HR-HPV subtype genetic testing. Exclusion criteria included individuals who had undergone hysterectomy for non-cervical cancer or non-cervical diseases. Informed consent was obtained from all research subjects. A total of 15,687 rural women underwent HR-HPV subtype genetic testing, distributed as follows: 3,235 from Bomi County, 2,795 from Chayu County, 1,271 from Motuo County, 4,612 from Gongbujiangda County, 50 from Lang County, and 3,724 from Bayi District (Figure 1). The Ethics Committee of the Third Affiliated Hospital of Guangzhou Medical University granted approval for this study.

The gynecological examination involved assessing the vagina, checking for visible abnormalities on the cervix, and conducting a bimanual pelvic examination. HR-HPV subtype gene detection was performed using the PCR fluorescence method, employing detection reagents for the 13 HR-HPV subtypes recommended by the World Health Organization (HPV types 16, 18, 31, 33, 35, 39, 45, 51, 52, 56, 58, 59, and 68). This allowed for clear identification of specific HR-HPV subtypes. The HPV infection rate, the infection rate of different HR-HPV subtypes, and the infection rate of HR-HPV subtypes among different age groups of rural women outside the Qinghai-Tibet Plateau were compared based on the reference (15).

### Statistical

Statistical analysis was conducted using Graphpad Prism 9.0 software. The age distribution of the research subjects conformed to a normal distribution, represented as x̄ ± s. The χ^2^ test was utilized to compare the HPV infection rates across various age groups and HR-HPV subtypes. A significance level of *α* = 0.05 was set for the comparison tests, and a *p*-value of <0.01 was considered statistically significant.

## Results

### Overview

Among the 15,687 eligible rural women, the average age was 46.02 ± 8.31 years. The largest proportion of participants was in the 35–39 age group, accounting for 20.78% (3,260/15,687), while the smallest proportion was in the 60–64 age group, accounting for 10.80% (1,694/15,687). The majority of the research subjects, 97.5%, were Tibetan, while the remaining participants were of Han ethnicity.

### Infection rate of HR-HPV among women of different age groups in rural areas of Nyingchi City, Tibet

The overall HR-HPV infection rate among women in rural areas was 13.00% (2,040/15,687). Among the different age groups, the 35–39 age group exhibited the highest infection rate of 15.31% (499/3,260), while the 50–54 age group showed the lowest infection rate of 9.69% (246/2,540). Notably, the HR-HPV infection rate decreased with increasing age, with the 55–59 age group having an infection rate of 12.23% (222/1,815), and the 60–64 age group having an infection rate of 10.92% (185/1,694). These differences in HR-HPV infection rates among age groups were found to be statistically significant (Table 1).

**Table 1:** HR-HPV infection status of women of different ages group in rural areas of Nyingchi City, Tibet.

Age Group (Years)	Cases	Number of infection	Infection rate(%)	*χ^2^*	*P*
35–39	3,260	499	15.31%	55.24	<0.0001
40–44	3,149	465	14.77%		
45–49	3,229	423	13.10%		
50–54	2,540	246	9.69%		
55–59	1815	222	12.23%		
60–64	1,694	185	10.92%		
Total	15,687	2040	13.00%		

HR-HPV, high-risk human papillomavirus.

### HR-HPV infection rate among women of different age groups betwee Nyingchi City, Tibet with rural areas outside the Qinghai-Tibet plateau

The overall HR-HPV infection rate in rural areas of Nyingchi City, Tibet was significantly higher at 13.00% (2,040/15,687) compared to rural areas outside the Qinghai-Tibet Plateau at 7.82% (9,249/118,237; *χ*^2^ = 635.7, *p* < 0.001). Specifically, when analyzing the HR-HPV infection rates in different age groups, including the 35–39, 40–44, 45–49, 50–54, and 55–59 age groups, the rates in rural areas of Nyingchi City, Tibet were significantly higher than those in rural areas outside the Qinghai-Tibet Plateau (χ^2^ = 253.0, 227.7, 114.0, 7.015, and 17.68, respectively, all *p* < 0.001). However, in the 60–64 age group, there was no significant difference in the HR-HPV infection rate between the two regions (*χ*^2^ = 1.622, *p* = 0.2028; Table 2).

**Table 2:** Comparison of HR-HPV infection among women of different ages in Nyingchi City, Tibet and rural areas outside the Qinghai-Tibet Plateau.

Age Group(Years)	Rural areas in Nyingchi City			Rural areas outside the Qinghai-Tibet Plateau			*χ^2^*	*P*
	Cases	Number of infection	Infection rate(%)	Cases	Number of infection	Infection rate(%)		
35–39	3,260	499	15.31%	25,322	1827	7.22	253	<0.0001
40–44	3,149	465	14.77%	26,248	1860	7.09	227.7	<0.0001
45–49	3,229	423	13.10%	26,304	2007	7.63	114	<0.0001
50–54	2,540	246	9.69%	19,698	1,604	8.14	7.015	0.0081
55–59	1815	222	12.23%	12,271	1,120	9.13	17.68	<0.0001
60–64	1,694	185	10.92%	8,394	831	9.90	1.622	0.2028
合计	15,687	2040	13.00%	118,237	9,249	7.82	635.7	<0.0001

HR-HPV, high-risk human papillomavirus.

### Infection rates of different HR-HPV subtypes among women In Nyingchi City, Tibet and rural areas outside the Qinghai-Tibet plateau

A total of 2,040 cases of HR-HPV infection were observed among women in rural areas of Nyingchi City, Tibet. The three most prevalent HR-HPV subtypes were HPV52 (20.15%, 411/2,040), HPV16 (12.45%, 254/2,040), and HPV58 (11.96%, 244/2,040). Similarly, among women in rural areas outside the Qinghai-Tibet Plateau, the top three HR-HPV subtypes were HPV52 (20.98%, 1,432/6,824), HPV16 (18.23%, 1,244/6,824), and HPV58 (11.06%, 755/6,824). The ranking of HR-HPV subtypes was consistent between the two regions. However, the HPV16 infection rate differed significantly at (12.45%, 254/2,040) in rural areas of Nyingchi City, Tibet and (18.23%, 1,244/6,824) in rural areas outside the Qinghai-Tibet Plateau (*χ*^2^ = 37.34, *p* < 0.001; Table 3).

**Table 3:** Comparison of infection status of different HR-HPV subtypes among women in Nyingchi City, Tibet and rural areas outside the Qinghai-Tibet Plateau.

HPV subtypes	Rural areas in Nyingchi City		Rural areas outside the Qinghai-Tibet Plateau		*χ^2^*	*P*
	1760		6,824			
	Number of infection	Infection rate(%)	Number of infection	Infection rate(%)		
HPV 16	254	12.45	1,244	18.23	37.34	<0.0001
HPV 18	138	6.76	509	7.46	1.119	0.2902
HPV 31	104	5.10	315	4.62	0.8101	0.3681
HPV 33	76	3.73	425	6.23	18.44	0.0001
HPV 35	74	3.63	215	3.15	1.132	0.2873
HPV 39	69	3.38	426	6.24	24.37	<0.0001
HPV 45	48	2.35	109	1.60	5.154	0.0232
HPV 51	195	9.56	472	6.92	15.75	<0.0001
HPV 52	411	20.15	1,432	20.98	0.6692	0.4133
HPV 56	102	5.00	306	4.48	0.9517	0.3293
HPV 58	244	11.96	755	11.06	1.263	0.261
HPV 59	148	7.25	209	3.06	71.41	<0.0001
HPV 68	177	8.68	407	5.96	18.77	<0.0001

HR-HPV, high-risk human papillomavirus; This table only lists the infection status of the 13 HR-HPV subtypes recommended by WHO.

### Infection rates of different HR-HPV subtypes among women of different age groups in rural areas of Nyingchi City, Tibet

The distribution of HR-HPV subtype infection varied among different age groups of women. In the 35–39 age group, the top three HR-HPV subtypes were HPV52, HPV16, and HPV58. Similarly, in the 40–44, 45–49, and 50–54 age groups, the predominant HR-HPV subtypes were HPV52, HPV16, and HPV58. Among women aged 55–59, the top three HR-HPV subtypes were HPV52 (16.22%, 36/222), HPV58 (10.36%, 23/222), and a combination of HPV51 and HPV16 (9.91%, 22/222). In the 60–64 age group, the most prevalent HR-HPV subtypes were HPV58 (15.14%, 28/185), HPV52 (13.51%, 22/185), and HPV59 (13.51%, 22/185; Table 4).

**Table 4:** Comparison of infection status of different HR-HPV subtypes among women of different ages in rural areas of Nyingchi City, Tibet.

HPV subtypes	35–39 Group		40–44 Group		45–49 Group		50–54 Group		55–59 Group		60–64 Group	
	499		465		423		246		222		185	
	Infection	Infection rate(%)	Infection	Infection rate(%)	Infection	Infection rate(%)	Infection	Infection rate(%)	Infection	Infection rate(%)	Infection	Infection rate(%)
HPV 16	65	13.03	61	13.12	54	12.77	34	13.82	22	9.91	18	9.73
HPV 18	35	7.01	29	6.24	27	6.38	18	7.32	18	8.11	11	5.95
HPV 31	21	4.21	20	4.30	21	4.96	15	6.10	14	6.31	13	7.03
HPV 33	16	3.21	14	3.01	17	4.02	12	4.88	11	4.95	6	3.24
HPV 35	15	3.01	16	3.44	12	2.84	10	4.07	12	5.41	9	4.86
HPV 39	22	4.41	16	3.44	14	3.31	7	2.85	5	2.25	5	2.70
HPV 45	10	2.00	8	1.72	9	2.13	6	2.44	10	4.50	5	2.70
HPV 51	50	10.02	41	8.82	42	9.93	25	10.16	22	9.91	15	8.11
HPV 52	113	22.65	103	22.15	95	22.46	39	15.85	36	16.22	25	13.51
HPV 56	24	4.81	21	4.52	18	4.26	14	5.69	15	6.76	10	5.41
HPV 58	57	11.42	58	12.47	48	11.35	30	12.20	23	10.36	28	15.14
HPV 59	30	6.01	26	5.59	31	7.33	17	6.91	19	8.56	25	13.51
HPV 68	41	8.22	52	11.18	35	8.27	19	7.72	15	6.76	15	8.11

## Discussion

Our survey revealed that the overall HR-HPV infection rate among women in rural areas of Nyingchi City, Tibet, was 13.00%, which was significantly higher than the infection rate among women in rural areas outside the Qinghai-Tibet Plateau (7.82%; *χ*^2^ = 635.7, *p* < 0.001). Furthermore, the infection rate in Nyingchi City was also significantly higher than the overall HR-HPV infection rate (9.19%) observed in Lhasa, Shigatse, and Nagqu in Tibet (15). Altitude was found to potentially impact the HPV infection rate, with Lhasa’s average altitude of 3,650 meters correlating to a 9.16% infection rate, and Shigatse’s average altitude of 3,836 meters correlating to an 8.86% infection rate (16). However, when compared with other regions, the overall HR-HPV infection rate among women in rural areas of Nyingchi City, Tibet, was lower than that reported in Shenzhen City (23.2%) (17), Shanxi Province (14.8%) (18), Taiwan (29.4%) (19), and certain areas of Hubei (20).

Regarding age groups, a “U”-shaped distribution pattern was observed in the HR-HPV infection rate (21), with the lowest rate recorded in the 50–54 age group at 9.69% (246/2540). The infection rate in women aged 35–54 showed a declining trend from 15.31 to 9.69%. Among women aged 55–59, the HR-HPV infection rate was 12.23% (222/1815), and among those aged 60–64, it was 10.92% (185/1694), indicating a consistent downward trend. This trend aligns with similar research findings on age distribution in other countries. A domestic study (22) similarly observed the phenomenon: the graphical representation of the positive infection rate of high-risk (HR) HPV follows a U-shaped function with respect to age. The peak occurs among females under 30 years old, with a second peak appearing among females over 50 years old. Women who are sexually active have a higher risk of HR-HPV infection compared to perimenopausal women (23, 24), while older adult women may experience a decline in immune function due to reduced postmenopausal ovarian sex hormone levels, increasing their susceptibility to HR-HPV infection or reducing their capacity for self-clearance of HR-HPV infection (25). Moreover, the HR-HPV infection rate among women in rural areas of Nyingchi City, Tibet, was significantly higher than that among women in rural areas outside the Qinghai-Tibet Plateau across all age groups, with a statistically significant difference (*p* < 0.001).

Previous studies have identified HPV types 16, 18, 31, 58, 52, 51, and 33 as the most common HR-HPV subtypes in the female population, with variations in their distribution and order of infection (26). In our study, the top three HR-HPV subtypes were HPV52 (20.15%, 411/2040), HPV16 (12.45%, 254/2040), and HPV58 (11.96%, 244/2040), which were consistent with the top three subtypes among women in rural areas outside the Qinghai-Tibet Plateau (12). Furthermore, our findings confirm that HPV52 is the most prevalent and common HR-HPV subtype in rural areas of China and even across Asia (26). Additionally, our study revealed a consistent distribution of the top three HR-HPV subtypes (HPV52, HPV16, and HPV58) among the 35–39, 40–44, and 45–49 age groups. In the 60–64 age group, the top three subtypes were HPV58 (15.14%, 28/185), HPV52 (13.51%, 22/185), and HPV59 (13.51%, 22/185).

In summary, our study provides valuable data on the composition and distribution of the top three HR-HPV subtypes among women in rural China and highlights the need for HPV infection prevention and the development of HPV vaccines in China. However, it is important to note that this study focused solely on rural areas of Nyingchi City and may not represent the overall situation in the Qinghai-Tibet region. Therefore, future research should employ rigorous study designs, expand the research scope, and increase the sample size for comprehensive investigation and analysis.

Furthermore, building upon this study, it would be beneficial to explore the relationship between HR-HPV subtype infection and cervical lesions and cervical cancer among women in rural areas of Nyingchi City, thus providing data support for reducing the incidence of cervical lesions and cervical cancer in this region.

However, the data collected exhibit limitations. Researchers have access to information such as marriage timing, commencement of sexual activity, the interval between screening and initial sexual activity, and childbirth history solely from Bomê County. Conversely, this data is unavailable for the remaining counties. Moreover, the data pertaining to low-risk HPV infection rates and multiple HPV infections in Nyingchi City is incomplete, with only partial data accessible from the Bayi District. Consequently, a thorough analysis and inclusion of comprehensive low-risk HPV infection rates and multiple HPV infections in this study’s outcomes are unfeasible, signifying a significant research gap and a regrettable limitation.

## Data availability statement

The original contributions presented in the study are included in the article/supplementary material, further inquiries can be directed to the corresponding author.

## Ethics statement

The experimental protocol was established, according to the ethical guidelines of the Helsinki Declaration and was approved by the Ethics Committee of the Third Affiliated Hospital of Guangzhou Medical University. All participants signed the informed consent.

## Author contributions

JL and XS: conceptualization, funding acquisition, and writing– review and editing. JL: formal analysis. XL: methodology. JL and XL: writing–original draft. All authors contributed to the article and approved the submitted version.

## Funding

This research was funded by Science and Technology Planning Project of Guangzhou (funding No. 202102010003); the Project for Key Medicine Discipline Construction of Guangzhou Municipality (No. 2021–2023-17) and Guangzhou Health Science and Technology Project (No. 20231A011091).

## Conflict of interest

The authors declare that the research was conducted in the absence of any commercial or financial relationships that could be construed as a potential conflict of interest.

## Publisher’s note

All claims expressed in this article are solely those of the authors and do not necessarily represent those of their affiliated organizations, or those of the publisher, the editors and the reviewers. Any product that may be evaluated in this article, or claim that may be made by its manufacturer, is not guaranteed or endorsed by the publisher.
